# Application of cognitive remediation in the world: new experiences from two schizophrenia rehabilitation centers in Togo and Benin

**DOI:** 10.1007/s00127-023-02603-z

**Published:** 2024-01-07

**Authors:** Giacomo Deste, Mawuko Kakli, Stefano Barlati, Gabriele Nibbio, Pacôme Dossou, Salomon Léonard Degila, Anna Ceraso, Jacopo Lisoni, Irene Calzavara-Pinton, Simona Villa, Antonio Vita

**Affiliations:** 1 Department of Clinical and Experimental Sciences, University of Brescia, Viale Europa 11, 25123 Brescia, Italy; 2 Department of Mental Health and Addiction Services, ASST Spedali Civili of Brescia, Brescia, Italy; 3 Association Saint Camille de Lellis, Lomé, Togo; 4 Diocese of Aného, Aného, Togo; 5 Congregation “Suore Misericordine” mission in Fatebenefratelli Hospital of Afagnan, Afanyagan, Togo

**Keywords:** Cognitive remediation, Evidence-based, Low-income countries, Mental health care, Psychosocial rehabilitation, Schizophrenia

## Abstract

**Purpose:**

People with schizophrenia in Sub-Saharan Africa often live in very difficult conditions, suffer important social isolation and usually do not receive any kind of treatment. In this context, some non-governmental initiatives have come to light, providing accommodation, food, primary healthcare, medications and, in some cases, education and rehabilitation. The aims of this study were to assess feasibility, effects, and acceptability of a Cognitive Remediation Therapy (CRT) intervention in the particular context of psychiatric rehabilitation in Togo and Benin.

**Methods:**

Patients diagnosed with schizophrenia accessing the “Saint Camille” association rehabilitation centers in Togo and Benin during the enrollment period were allocated consecutively with a 1:1 proportion to receive a manualized CRT intervention (46 one-hour sessions over 14 weeks) or continuing Treatment As Usual (TAU). The assessment included validated measures of cognitive performance and real-world functioning and was performed at baseline and at the conclusion of treatment.

**Results:**

All subjects that were invited into the study agreed to participate and completed the intervention, for a total of 36 participants. CRT produced greater improvements than TAU in processing speed, working memory, verbal memory, cognitive flexibility, and executive functions measures, with moderate to large effect sizes, in particular in processing speed and working memory domains.

**Conclusions:**

CRT represents a feasible and effective psychosocial intervention that can be implemented even in contexts with very limited resources, and could represent an important instrument to promote the rehabilitation process of people living with schizophrenia in low-income countries.

## Introduction

### Background

Schizophrenia is a severe mental disorder that is frequently associated with cognitive impairment, encompassing both neurocognition [1] and social cognition [2], and poor functional outcomes [3–5].

Schizophrenia has a global prevalence of almost 1%; although slightly different prevalence rates are reported in some countries, probably as a consequence of differences in diagnostic classifications and availability of mental health services resources, this estimate appears to be highly consistent on a worldwide level [6–8].

In Sub-Saharan Africa, people living with severe mental disorders, and with schizophrenia spectrum disorders (SSD) in particular, often live in very difficult conditions [9–11]. They are subject to high levels of stigma: psychotic symptoms are often interpreted as a sign of demonic possession, a curse, the work of malign spirits, or as a punishment for the sins of the afflicted [12–14]. In this context, people with schizophrenia often live in a condition of abandonment and severe isolation. As a result, they are often forced to homelessness and wandering, and in some cases they are imprisoned and restrained for long periods of time [15, 16].

In Togo and Benin, and more generally in most countries of the region, access to treatment for people with severe mental disorders is severely lacking: recent estimates report that 76–85% of patients do not receive any kind of treatment. This is mostly due to the lack of professionals and resources dedicated to mental health care, also because local public health policies tend to favor investments in infectious diseases and malnutrition treatments [17, 18]. In fact, both Togo and Benin feature only a single hospital center specifically dedicated to mental disorders treatment, in both cases with a capacity slightly under 180 beds, despite the large population of both countries (8,500,000 people in Togo and 12,500,000 in Benin) [19]. In most cases, due to the lack of any structured outpatient service, people seeking help for mental disorders are treated by traditional healers: while some healers genuinely attempt to provide assistance, this results in additional difficulties in receiving evidence-based treatments [9, 20]. In other cases, traditional support is completely detrimental: the so called “prayer camps”, for instance, appear to be places of detention, where people living with SSD not only do not receive any appropriate treatment, but are also restrained and forced to live in unhygienic and problematic conditions [15]. The lack of appropriate treatments for long periods of time, combined with high levels of stigma and social isolation, contribute to determine severe clinical conditions, with important breakdown of psychosocial functioning. In this context, some private, non-governmental initiatives have come to light, providing accommodation, food, primary health care, medications and, in some cases, education and rehabilitation. These initiatives are few and are developing mostly thanks to funding provided by Religious Institutions, but in recent years have been able to grab the attention of many people seeking for mental health care, who could not afford other, more costly treatments. In this situation, implementing treatments beside pharmacological therapy that are effective on core dimensions of schizophrenia and that can be delivered also with limited resources is of paramount importance.

Cognitive remediation (CR) is an evidence-based intervention that is effective in improving both cognitive abilities and real-world functioning in people living with schizophrenia [21–24] and is characterized by a good acceptability profile [25] and good cost-effectiveness [26, 27]. In particular, pen-and-paper interventions, which are as effective as computerized ones [28] , require only an active and trained therapist to be implemented effectively in clinical practice, and, given this low cost requirement, may represent a very valuable treatment in rehabilitation settings in developing countries. According to recent literature, CR also appears to be particularly effective in more clinically compromised participants [28, 29] this fact also suggests that it could represent a really valuable intervention in countries such as Togo and Benin and Sub-Saharan Africa in general, considering that the clinical condition of patients in this context is often severe and characterized by cognitive and psychosocial deterioration.

Due to the need of effective evidence-based rehabilitation interventions in Sub-Saharan Africa, one of the Authors (MK), who was born in Benin and is currently working there as a member of a non-governmental association, decided to move to Italy in 2018, to attend the course of Techniques of Psychiatric Rehabilitation at the University of Brescia. During the course of his studies, he traveled back and forth several times from Italy to Togo and Benin, where he provided teaching and training to local mental healthcare workers on rehabilitation interventions and CR, thus preparing the field for the design and implementation of the present pilot study.

### Aims

The aims of the present pilot study were to assess feasibility, effects, and acceptability of a CR intervention in the context of psychiatric rehabilitation in Togo and Benin. Considering the particular context of care, subjective experiences and individual perspectives of therapists and participants were also be investigated.

## Materials and methods

### Study design

This is a pilot controlled study comparing cognitive and functional outcomes of two groups of inpatients diagnosed with schizophrenia, hospitalized in two psychiatric health centers in Togo and Benin. The study took place from February 2022 to October 2022.

### Context of care

Psychiatric health centers where this study was conducted are part of the “Saint Camille” non-governmental association. The “Saint Camille” association was founded in Ivory Coast in 1983 by Gregoire Ahongbonon to provide assistance to psychiatric inpatients in a university hospital and to provide food and shelter for people with severe mental disorders, which in this country are frequently homeless and abandoned by their families. The first psychiatric treatment facilities run by the association were opened in 1993, while in 2004 the first center was opened in Benin, and in 2015 another was founded in Togo. At present, three inpatient assistance and care centers are open in Togo and six in Benin. The psychiatric centers now represent consolidated and renowned reference point for people living with mental disorders in Togo and Benin. Access to the centers is direct and free of charge: patients can access also accompanied by their family or by law enforcement officials during compulsory admissions. In some cases, healthcare workers of the association rescue abandoned patients in the country and welcome them in the centers. Each center hosts between 150 and 200 patients usually aged between 18 and 30 years with a wide range of diagnoses, most frequently SSD, bipolar disorder, personality disorders, depression, anxiety, and substance use disorders. For people diagnosed with schizophrenia, pharmacological treatment consists primarily of first-generation antipsychotics: given the low local purchasing power and the fact that patients often cannot independently afford them, second generation molecules are usually unavailable. Mental healthcare workers, employed in the centers, include nurses, psychologists, and professional educators. In some occasions, trained stabilized patients directly contribute to rehabilitation activities, working in the centers as mental health care support personnel. Overall, in 2019, a total of 1949 patients were treated in Togo and 4500 in Benin. In 2020, 2100 patients were treated in Togo and 5057 in Benin.

### Inclusion and exclusion criteria

Inclusion criteria were: (I) clinical diagnosis of schizophrenia according to DSM-5 criteria [30], (II) clinical stability, defined as the absence of major changes in pharmacological treatment molecule or dosage in the previous month, and (III) informed consent to participate in the study, documented by signing a dedicated form.

Diagnoses were performed by a medical doctor with several years of experience in the treatment of mental disorders in rehabilitation centers, with the support of a psychiatrist when required.

Exclusion criteria were: (I) history of neurological disease or major head trauma, (II) diagnosis of intellectual disability, and (III) diagnosis of alcohol or substance use disorders.

Participants were invited to join the study during a visit in which the study protocol and the interventions were presented, also discussing daily-life problems related to cognitive difficulties.

Participants were allocated consecutively with a 1:1 proportion to two groups, one receiving the Cognitive Remediation Therapy (CRT) intervention and one continuing Treatment As Usual (TAU).

All patients were treated with stable antipsychotic treatment.

### Interventions

#### Cognitive remediation therapy

CRT [31] is a behavioral training-based psychosocial intervention that aims to enhance basic mental abilities through the use of non-emotionally relevant material, to facilitate more complex social behaviors and daily life functioning. CRT specifically targets cognitive functions that are impaired in schizophrenia, such as attention, memory, and executive functions. A trained therapist and the participant are involved in face-to-face sessions, in which the use of strategies and learning techniques, such as errorless learning, scaffolding, and massed practice, are constantly implemented, with a high level of personalization of the tasks during the whole program. The effectiveness of this specific CR intervention has been well documented [32, 33]. CRT intervention has been specifically chosen for this study given its feasibility and adaptability to the context of care of the psychiatric centers in Togo and Benin. The program was composed by 40 one-hour sessions, delivered by the active and trained therapists three times a week during a period of approximately 14 weeks. The CRT intervention was completely carried out in a pencil-and-paper manner and did not involve any computer exercise.

The CRT intervention was conducted following a manualized approach [31] and was composed by three modules: “Cognitive flexibility”, “Memory”, and “Planning”. The “Cognitive flexibility” module is composed by exercises that involve focusing and shifting the attention between tasks, while keeping specific information memorized (e.g. selecting only even numbers in a list). The “Memory” module is focused on working memory, verbal memory and visual memory, with specific and dedicated exercises. The “Planning” module requires a growing number of instructions to memorize and use to fulfill defined objectives: this module is focused in particular on creating and applying cognitive strategies to reach specific goals.

The difficulty of the tasks can be scaled according to the participant’s performance, and increases with the progression of the exercises; the whole intervention is based on the principles of errorless learning, cognitive scaffolding and massed practice.

#### Treatment As Usual

Patients in the Treatment As Usual (TAU) group participated in additional sessions of rehabilitation activities, delivered with similar intensity and duration compared to CRT sessions, to represent a more appropriate control group. These activities were chosen among those usually delivered in psychiatric centers to all patients, including leisure activities, art, work (gardening, agriculture, farming) and professional training (worker, mechanic, blacksmith), house-keeping, cooking, support in centers routine and maintenance, as well as sessions of other non-cognitive oriented psychosocial interventions. These activities were supported by trained professionals and were delivered in a perspective of progressive independence.

Patients allocated in TAU group were also placed in a waiting list to receive CRT treatment.

### Training of therapists

For the present study, the CRT manual [31] has been adapted in French by a native French speaking author (KD). Back translation of the CRT manual was then performed by two other authors (GN and SV).

Mental healthcare workers of the “Saint Camille” non-governmental association, working in the centers of Togo and Benin, were specifically trained by KD to deliver the CRT intervention. Training was attended by all the professionals that subsequently performed the assessment or delivered the interventions and included multiple frontal lessons, practical sessions and simulations of real-world situations for all assessment tools as well as all modules of the CRT program. Training sessions were delivered by KD and were directly supervised by GD through multiple online video conferences.

### Assessment

Participants were assessed at baseline (T0) and at the conclusion of the interventions (T1) using dedicated measures of cognitive performance and psychosocial functioning. The assessment was performed by trained psychologists that were not directly involved in delivering CRT intervention and were blind regarding participants allocation into intervention arms.

Cognitive assessments included different tasks measuring performance in key cognitive domains: the Trail Making Test-A and B (TMT-A and B) [34], the Wechsler Adult Intelligence Scale—Revised (WAIS-R) Digit Span Backward and Digit Symbol Coding [35], and the Rey Auditory Verbal Learning Test (RAVLT) [36]. As no definite scientific consensus is available regarding the cognitive domains explored by each specific test, test performances were attributed to different cognitive domains according to previous meta-analytic literature [28, 37]: TMT-A: Processing Speed; TMT-B: Working Memory; TMT B-A: Executive Functions and Cognitive Flexibility; WAIS-R Digit Span Backward: Working Memory; WAIS-R Digit Symbol-Coding: Processing Speed; RAVLT: Verbal Learning and Memory. Raw scores of the single tests were included in the analyses.

Real-world psychosocial functioning was measured with the Global Assessment of Functioning (GAF) [38]. The GAF is a frequently-used, simple and comprehensive instrument recommended for the assessment of social, occupational and psychosocial functioning [39]; overall score ranges from 0 to 100, with higher scores representing better functioning.

Inter-rater reliability of professionals performing the assessment was evaluated for all instruments in a dedicated simulation session that took place at the end of the training program and was found to be good (interclass coefficient correlation above 0.8 for all measures).

### Statistical analyses

Baseline comparisons between the CRT and TAU groups were performed to assess potential differences in demographic and clinical factors that could act as moderators of treatment effects using independent samples *t* tests for continuous variables and chi-squared tests for categorical variables. Variables that emerged as significantly different at baseline between groups were introduced as covariates in the between-groups treatment effect analyses.

Within-group changes in treatment outcomes measures were assessed comparing baseline and post-treatment scores (T0–T1) using paired samples *t* tests. Between-groups changes in treatment outcomes measures at the end of the active treatment phase (T1) were assessed using analysis of covariance (ANCOVA), covaried by baseline values (T0) to account for pre-intervention differences and education years. Between-groups effects sizes were also calculated as Cohen’s D [40].

Statistical analyses were performed using SPSS version 15.0; *p* values < 0.05 (two tailed) were considered statistically significant.

### Acceptability and personal impressions

Being a pilot study, with a specific focus on feasibility and acceptability of the intervention, the subjective impressions regarding CRT of some therapists and of some participants allocated to the CRT arm have been collected and reported in a narrative way, to better represent the characteristics of the intervention in this very particular context of care.

## Results

### Characteristics of the sample

All subjects that were invited into the study agreed to participate, provided informed consent and completed both the interventions and the assessments. The total sample was composed by 36 participants, M:F = 27:9, mean age 31.1 ± 8.5 years. Twenty participants were recruited in Togo and 16 in Benin. The whole sample had a mean education of 10.3 ± 2.3 years, and mean duration of illness was 10.6 ± 15.8 years; 16 participants were unemployed. The TAU group had fewer years of education at baseline, so this variable was included as covariate in the between-groups treatment effect analyses. No other between-group difference emerged regarding baseline socio-demographic characteristics. In addition, no baseline differences emerged comparing participants from Togo and Benin. Sample characteristics and baseline comparisons for socio-demographic variables are reported in Table 1. Significant between-group differences were observed concerning baseline cognitive variables, with CRT group performing better in all tests (*p* < 0.05) except the WAIS-R Digit Span Backward (*p* = 0.053). These effects were accounted for by including baseline values as covariates in ANCOVA comparisons.

**Table 1:** Between-groups comparisons for socio-demographic and clinical characteristics

Variable	Total sample (*N* = 36)	CRT (*n* = 18) Mean ± DS	TAU (*n* = 18) Mean ± DS	*t* test/*χ*^2^ test	*p *value
Age (years)	31.08 (± 8.47)	29.78 (± 9.56)	32.39 (± 7.27)	− 0.923	0.363
Gender (M:F)	27:9	14:4	13:5	0.148	0.500
Education (years)	10.33 (± 2.34)	11.50 (± 2.26)	9.17 (± 1.82)	3.413	0.002**
Duration of illness (years)	10.65 (± 15.82)	8.13 (± 11.21)	14.70 (± 21.36)	− 1.032	0.312
Employment (employed:unemployed)	20:16	10:8	10:8	0	1.000
Center (Togo:Benin)	20:16	10:8	10:8	0	1.000

*CRT* cognitive remediation therapy, *TAU* treatment as usual

*p < 0.05; **p < 0.01

### Effects of the intervention

Considering the within-group baseline to post-treatment analyses (T0–T1), a significant improvement in all neuropsychological measures and in psychosocial functioning was observed in the CRT group. On the contrary, no significant change in any treatment outcome measure was observed in the TAU group.

Several between-groups treatment effects were observed in ANCOVA analyses (post-treatment covaried by baseline and education years). CRT produced greater improvements than TAU in processing speed, working memory, verbal memory, cognitive flexibility, and executive functions measures, with moderate to large effect sizes, in particular in processing speed and working memory domains. No significant between-group effect was observed for psychosocial functioning as measured by GAF scale.

Results of within- and between-groups analyses are reported in Table 2.

**Table 2:** Effects of CRT on cognitive and functional outcomes

Variable	CRT T0 (*n* = 18) Mean ± DS	TAU T0 (*n* = 18) Mean ± DS	CRT T1 (*n* = 18) Mean ± DS	TAU T1 (*n* = 18) Mean ± DS	CRT T0vsT1 (*n* = 18) *p *value	TAU T0vsT1 (*n* = 18) *p *value	*ANCOVA* *F*	*ANCOVA* *p *value	Effect size (Cohen’s* D*)
TMT-A (processing speed)	94.89 (± 39.51)	133.06 (± 55.52)	73.61 (± 26.54)	140.11 (± 57.89)	0.001**	0.397	11.874	0.002**	0.58
TMT-B (working memory)	213.78 (± 115.48)	298.22 (± 102.45)	156.61 (± 60.77)	316.22 (± 110.95)	0.011*	0.112	17.220	< 0.001**	0.67
TMT B-A (executive functions/cognitive flexibility)	118.89 (± 87.21)	165.17 (± 61.52)	83.00 (± 41.71)	176.11 (± 77.11)	0.047*	0.462	8.081	0.008**	0.61
WAIS-R digit symbol coding (processing speed)	27.78 (± 8.80)	20.22 (± 7.70)	36.22 (± 10.78)	20.67 (± 8.87)	< 0.001**	0.684	10.329	0.003**	0.94
WAIS-R digit span backward (working memory)	13.89 (± 2.05)	11.89 (± 3.69)	19.00 (± 4.16)	11.72 (± 4.18)	< 0.001**	0.779	17.030	< 0.001**	1.73
RAVLT (verbal learning and memory)	12.17 (± 2.43)	10.12 (± 2.45)	14.39 (± 1.19)	10.71 (± 2.82)	< 0.001**	0.163	12.117	0.002**	0.65
GAF (psychosocial functioning)	61.00 (± 3.80)	55.39 (± 3.24)	62.06 (± 4.19)	55.61 (± 3.43)	0.001**	0.331	2.034	0.163	0.23

ANCOVA covariates include T0 scores and education (years)

*CRT* cognitive remediation therapy, *GAF* global assessment of functioning, *RAVLT* Rey auditory verbal learning test, *TAU* treatment as usual, *TMT* trial making test, *WAIS-R* Wechsler adult intelligence scale—revised

*p < 0.05; **p < 0.01

### Acceptability and personal impressions: therapists

*Therapist 1* remarked the novelty of the intervention and was surprised of its feasibility considering the few economic resources required to implement the program. He also highlighted the need of therapists’ commitment and motivation: to complete the program, therapists must be ready to deal with a number of unexpected limiting events, as well as with participant’s initial possible frustration for unsuccess. He reported how participants motivation progressively developed as the program went on. He was able to observe changes in participants’ daily routine, with improvements in self-care and work activities. An improvement in communication abilities, social skills, and active participation in group talking was also observed by the Therapist, who was pleased to be acquainted with participants, by experiencing their personal talents and their wishes, thus eventually helping some of them to express themselves in different contexts.

*Therapist 2* reported that CR promoted the establishment of a therapeutic relationship between therapists and participants, leading to an improvement in self-confidence. He also reported improvement in participants ability to express their own cognitive and personal difficulties.

*Therapist 3* reported that the program is characterized by a strict and steady contact with the participants, allowing an immediate observation of clinical changes and problems, such as pharmacological side effects, that can emerge during CRT sessions.

### Acceptability and personal impression: participants

*Participant 1* (V.K.S., male, age 21 years) found that numerical exercises and visual tasks helped him starting over to draw, and, therefore, also in expressing himself. He reported that this was related to an increased sense of self-confidence in planning the work and using materials. He was also gratified by family members, who explicitly pointed out his renewed commitment in house-keeping activities. He now plans to eventually return to school.

*Participant 2* (I.I., male, age 24 years) reported some initial difficulties in facing CR tasks due to frequent jumping to conclusion bias. Thanks to CRT, he eventually learned how to prevent impulsive reactions and to plan his responses by looking for strategies. This improved his school performance to the point that he wanted to teach the use of these strategies to a friend. Finally, he developed personal strategies to face daily problems: for instance, he bought a clock to help him in organizing his daily routine.

*Participant 3* (K.K., male, age 54 years) previously worked as a teacher, but he could not work anymore due to his illness. He reported an increase in subjective intrinsic motivation related to CRT sessions participation, which was related to an increasing ability to complete CR tasks. He reconnected with some old friends, and he now enjoys participating in group conversations with family members and friends. He also reported subjective improvements in time management and better multitasking performance.

## Discussion

The results of the present study attest that CR intervention is feasible in the context of psychiatric centers of Togo and Benin. To the best of our knowledge, this study represents the first experience of a structured implementation of a CR intervention in such a context of care. This result is of particular interest as it opens new treatment perspectives, which consider the implementation of non-pharmacological, low-cost interventions in low-income countries.

The CRT intervention emerged as superior to TAU in most outcomes. In fact, a significant improvement in different cognitive domains was reported only in the CRT group, whereas no benefits were observed in the TAU group. This result is in line with recent scientific evidence underlining the positive effects of CR in improving cognitive performance, especially when the interventions are implemented into an integrated rehabilitation program [22, 28, 41–44]. Observed effect sizes were considerably larger than those reported in most meta-analytic works [21, 24, 28, 45]: this could be due to the specific and unique features of treatment setting and of the included sample, characterized by participants with severe forms of the disorder, with long duration of untreated psychosis and with high levels of social difficulties, which could highly benefit from the treatment [46, 47].

Surprisingly, no significant treatment-related effect was observed for psychosocial functioning. This could also be due to the peculiarities of the context, where opportunities to engage in novel activities may be more limited than in high-income contexts with more available resources, and to the lack of a follow-up observation that could better detect changes in this area.

Subjective experiences of therapists and participants are also of interest. In particular, the subjective benefits reported by participants hint towards a substantial increase in intrinsic motivation, which represent an important component of treatment effectiveness [48, 49].

Some limitations have to be taken into account. The explorative and preliminary nature and the focus on feasibility of the present pilot study are associated with a small sample size, and the allocation to the groups did not follow a fully randomized procedure: both these issues limit the generalizability of the results. Due to the limited resources available in the centers, it was not possible to provide therapists with clinical assessment tools to evaluate symptom severity, which could have provided important additional information. Likewise, no assessment of the impact of pharmacological treatment, of other moderator effects, or of social cognition performance could be carried out. Finally, no follow-up assessments were included in the study.

All patients completed the intervention, meaning that they completed at least 80% of sessions. However, data on the average number of attended sessions, on which sessions were lost by each participant and on the causes of absence were not recorded and are not currently available.

Finally, all patients accessing the centers were consecutively allocated in the intervention groups without any prior selection: in this regard, participants in the study can be considered as representative of patients accessing the centers. However, while the centers implement an open and inclusive policy and provide care to patients accessing them, it cannot be completely ruled out that patients accessing the centers may represent a subgroup of individuals with an overall better psychosocial functioning profile, compared to other individuals living with schizophrenia in the region.

Despite these limitations, the results of the present study show that even in a context of care characterized by very limited economic resources dedicated to mental health, CR may represent a feasible and effective therapeutic asset for people living with schizophrenia, suggesting that CR could be systematically implemented in rehabilitation programs in addition to other non-pharmacological interventions.

Future developments of the present line of work include replicating the results in a randomized trial and implementing a wider panel of assessment tools. Moreover, including follow-up observations could provide further interesting information.

## Conclusions

CR interventions appears to be highly feasible and to provide substantial improvements in cognitive performance for patients living with schizophrenia in the context of care of west Sub-Saharan Africa. Considering the results of the present study and the important benefits provided for participants, implementing evidence-based rehabilitation interventions that are characterized by low cost and low resource requirements in clinical practice could truly represent a game changer for the lives of people with schizophrenia in low-income regions of the world.

## Data Availability

Data supporting the findings of the present study are available from the Corresponding Author upon reasonable request.
